# The Liver Frailty Index predicts survival in systemic therapy for hepatocellular carcinoma: a multicentre prospective cohort study

**DOI:** 10.1016/j.esmogo.2024.100043

**Published:** 2024-02-20

**Authors:** K.M.J. Waller, D.S. Prince, E.H.Y. Lai, M.T. Levy, S.I. Strasser, G.W. McCaughan, M.L.P. Teng, D.Q. Huang, K. Liu

**Affiliations:** 1 AAW Morrow Gastroenterology and Liver Centre, Royal Prince Alfred Hospital, Camperdown; 2 School of Public Health, Faculty of Medicine and Health, University of Sydney, Camperdown, Australia; 3 Central Clinical School, Faculty of Medicine and Health, University of Sydney, Camperdown, Australia; 4 Department of Gastroenterology and Hepatology, Liverpool Hospital, Liverpool; 5 South West Sydney Clinical School, Faculty of Medicine and Health, University of New South Wales, Liverpool, Australia; 6 Department of Radiology, Royal Prince Alfred Hospital, Camperdown; 7 Centenary Institute, The University of Sydney, Camperdown, Australia; 8 Division of Gastroenterology and Hepatology, National University Hospital, Singapore; 9 Department of Medicine, Yong Loo Lin School of Medicine, National University of Singapore, Singapore

**Keywords:** frailty, hepatocellular carcinoma, liver cancer, systemic therapy

## Abstract

**Background:**

Systemic therapy for hepatocellular carcinoma (HCC) can prolong survival, but outcomes vary, and predictors of response are not fully defined. Frailty is associated with worse outcomes in cirrhosis and liver transplantation, but its impact on patients with advanced HCC is unknown.

**Patients and methods:**

An international, multicentre, prospective, observational cohort of adults commencing systemic therapy for HCC from 2019 to 2022 was analysed. Frailty was assessed by the Liver Frailty Index (LFI). The primary outcome was overall survival; secondary outcomes were disease progression, adverse events, and treatment discontinuation.

**Results:**

Among 102 patients, 80% were male and the median age was 67 years [interquartile range (IQR) 60-73 years]. Most had viral hepatitis (hepatitis C virus 39%, hepatitis B virus 29%), were Child–Pugh A (75%), Eastern Cooperative Oncology Group (ECOG) 0-1 (89%), and Barcelona Centre Liver Cancer (BCLC) stage C (59%). Similar proportions received tyrosine kinase inhibitors (54%) and immunotherapy (46%). The median LFI was 4.13 (IQR 3.81-4.43): 4% were robust (LFI <3.2), 75% were pre-frail (LFI 3.2-<4.5), and 22% were frail (LFI 4.5+). LFI was independently associated with death (adjusted hazard ratio 1.74, 95% confidence interval 1.17-2.59, *P* = 0.006), after adjustment for Child–Pugh score and albumin–bilirubin grade. The optimal cut-off for survival at 1 year was LFI 4.2 (area under the curve 0.658), significant on univariable and multivariable analyses at predicting death. Frailty was associated with earlier systemic therapy discontinuation, despite similar rates of disease progression and adverse events; cessation of treatment due to functional decline was more common among frail patients. Sensitivity analysis excluding patients above Child–Pugh B8 or ECOG 2 did not change results.

**Conclusion:**

The LFI is an independent predictor of death among patients with HCC undergoing systemic therapy.

## Highlights

• Frailty is associated with mortality of patients with HCC undergoing systemic therapy.
• Frailty is also associated with earlier cessation of systemic therapy.
• There is no association of frailty with adverse events or disease progression.
• Optimal frailty cut-offs in HCC remain to be defined.

## Introduction

Liver cancer is globally the fifth most common cancer diagnosed, and the third most common cause of cancer-related death.^1^ Hepatocellular carcinoma (HCC) accounts for 80% of global liver cancers,^2^ with deaths from HCC modelled to increase 55% by 2040.^3^ A substantial proportion of patients with HCC are diagnosed at an advanced stage, where systemic therapy is recommended.

The combination of atezolizumab and bevacizumab is now the first-line therapy for those with advanced [Barcelona Centre Liver Cancer (BCLC) stage C] or intermediate multinodular HCC (BCLC stage B) not amenable to locoregional therapy.^4^ Tyrosine kinase inhibitors (TKIs), such as sorafenib and lenvatinib, remain alternative options for those intolerant or contraindicated to immunotherapy, in resource-limited settings where infusional therapies are unavailable, or as per patient preference. Given the significant side-effects and variable response rates, identifying those candidates who have favourable risk/benefit profiles for systemic therapy is an area of need. The BCLC staging criteria recognise unsuitability for systemic therapy among those with decompensated cirrhosis (Child–Pugh score C) and poor performance status [Eastern Cooperative Oncology Group (ECOG) over 2].^4^ Much effort has been spent to identify additional prognostic factors to optimise patient selection for HCC systemic therapy.^5^^,^^6^

Frailty is increasingly recognised as an important prognostic marker for patients with advanced liver disease.^7^ Specifically, the Liver Frailty Index (LFI) is a validated, objective bedside test, developed at the University of California, San Francisco,^8^ which has been shown to predict survival among liver transplant (LT) waitlisted patients,^9^, ^10^, ^11^ and decompensation and hospital readmission among cirrhosis patients.^12^^,^^13^ The LFI is easy to learn, takes 3-5 min to carry out with minimal equipment (dynamometer), and has good interobserver reliability.^14^ It can be calculated online from three metrics: dominant hand grip strength, time to do five chair stands, and seconds holding balance in three positions. The LFI is a continuous variable: among the index cohort, the median LFI was 4.0. In addition, patients are categorised as frail (LFI 4.5+), pre-frail (LFI 3.2-<4.5), or robust (LFI <3.2), representing the bottom 20%, middle 60%, and top 20% of patients with cirrhosis awaiting LT.^8^^,^^15^

Frailty is well recognised in oncology as a predictor of outcomes of systemic therapy, particularly among older patients, for whom frailty predicts worse mortality and increased adverse effects from chemotherapy.^16^ Limited studies suggest a high prevalence of frailty in HCC,^17^ although among transplant waitlisted patients, those with HCC were more robust than other candidates, with a median LFI of 3.2.^18^ Prospective studies examining the association of frailty and survival in people with HCC, especially advanced HCC, are lacking. One study of nutritional markers in 56 patients with HCC included only a single patient receiving systemic treatment; they found that LFI was not an independent predictor of survival, but noted that a deterioration in LFI was associated with worse complication-free survival.^19^ At earlier cancer stages, other frailty indices have shown an impact on HCC outcomes: the clinical frailty scale (a subjective 10-point scale) impacted survival after hepatectomy for HCC,^20^ and the modified frailty index (functional status plus selected comorbidities) predicted survival after transarterial chemoembolisation.^21^ A registry study using the hospital frailty risk score [based on the International Classification of Diseases, 10th Revision (ICD-10) codes] showed that more patients hospitalised for HCC were frail (58%) than non-frail (42%), with frailty shown to independently predict in-hospital mortality (unable to account for liver function or cancer stage).^22^

The prognostic value of frailty has not been studied among patients with advanced HCC, which formed the aim of this multicentre study.

## Patients and methods

We conducted a prospective, multicentre, international, observational cohort study of all patients commencing systemic therapy for HCC at least 18 years of age, from 2019 to 2022 at three sites: Royal Prince Alfred Hospital (RPAH), Camperdown, Australia; Liverpool Hospital, Liverpool, Australia; and the National University Hospital (NUH), Singapore. Patients were excluded if they had a previous LT, or frailty was not recorded within 3 months of starting therapy [noting the impact of coronavirus disease 2019 (COVID-19) pandemic on face-to-face appointments]. Ethics approval was obtained via the Royal Prince Alfred Hospital Human Research Ethics Committee, protocol X20-0158, 2020/ETH00683, and 2020/STE01533, including a waiver of patient consent.

The decision to commence systemic therapy was made by the treating physician, supported by a multidisciplinary team. Some patients were treated outside of criteria in the approved product information. Choice of systemic therapy was decided by the treating physician after informed patient discussion, and reflected available options over the time course of this study (including clinical trials). For instance, in Australia, lenvatinib was publicly funded in March 2019, and atezolizumab–bevacizumab in November 2020.

The LFI, described earlier, was calculated by clinical nurse consultants at each site at the commencement of systemic therapy. All sites followed the same method for carrying out the LFI and used the same model to measure grip strength. Frailty was recorded and analysed both as a continuous score (i.e. LFI) and category (i.e. frail, pre-frail, robust according to pre-defined cut-offs, described earlier).

Baseline data collected included patient demographics [age, sex, body mass index (BMI)], underlying liver disease aetiology and severity [Child–Pugh score, Model for End-stage Liver Disease (MELD) score, albumin–bilirubin (ALBI) grade], tumour stage [BCLC stage, α-fetoprotein (AFP)], and performance status (ECOG). Baseline sarcopenia data were collected retrospectively for a subset of patients with available computed tomography scans, using the cross-sectional area of the psoas muscle at level L3, height- and sex-adjusted, as previously described.^23^

The primary outcome was overall survival from the time of commencement of systemic therapy. Secondary outcomes were disease progression, defined by the Response Evaluation Criteria in Solid Tumors version 1.1,^24^ adverse events, graded according to the Common Terminology Criteria for Adverse Events version 5.0,^25^ and drug discontinuation for >30 days. Descriptive data were collected regarding reasons for therapy discontinuation (disease progression, adverse effects, patient preference, decompensation, and functional decline) and subsequent therapies after cessation. Clinicians were not blinded to frailty status or results.

Characteristics were compared between groups using chi-square test for categorical variables and Student’s *t*-test for continuous variables. For time to event analysis, univariable effects were estimated using Cox proportional hazards, and significant factors (at α = 0.10) incorporated into a multivariable Cox proportional hazards model, using backwards stepwise regression. Patients were censored at last follow-up. Multicollinearity between covariates was assessed with a variance inflation factor with values exceeding 5 considered significant. Frailty was analysed as both a continuous variable and by the pre-defined categories. Given that the pre-defined frailty categories have not been validated in HCC, the primary outcome (survival) was compared at different LFI cut-offs using log-rank score. Receiver operator curve analysis was used to determine the optimal cut-off of LFI to predict survival at 1 year, using the nearest to (0,1) criteria. Sensitivity analyses were carried out to test the robustness of results by excluding patients with Child–Pugh score B9 and higher and/or ECOG 3. For the subset of patients with available data, sarcopenia was analysed as a categorical and continuous variable in the same fashion; correlation between frailty score and sarcopenia was assessed using Spearman’s rank correlation coefficient. All statistical analysis was carried out using Stata 14.2 (StataCorp, College Station, TX).

## Results

### Cohort characteristics

After excluding 11 patients due to inadequate frailty data, 102 patients were included (Table 1), 47 from RPAH, 35 from Liverpool, and 20 from NUH, with 103.6 person-years of follow-up. Most were male (82), with a median age of 67 years [interquartile range (IQR) 60-73 years]. The most common aetiologies of liver disease were viral hepatitis for 70 patients (40 hepatitis C virus, 30 hepatitis B virus), alcohol-related cirrhosis (28), and non-alcoholic steatohepatitis (21). Twenty-four patients had multiple causes of liver disease. Most patients had Child–Pugh A cirrhosis (75%) and had good performance status (ECOG 0-1 89%). BCLC stages were B (38%), C (59%), and D (3%). Similar proportions were treated with TKIs (lenvatinib 39%, sorafenib 15%) and immunotherapy (39% atezolizumab–bevacizumab, 7% other immunotherapy).

**Table 1:** Baseline characteristics of patient cohort based on frailty status

Characteristics, *n* (column %)	Total	Robust	Pre-frail	Frail	*P*
Total (row %)	102	4 (4)	76 (75)	22 (22)	
Sex					0.354
Male	84 (82)	3 (75)	65 (86)	16 (73)	
Female	18 (18)	1 (25)	11 (14)	6 (27)	
Age (years), median (IQR)	67 (60-73)	56 (40-71)	66 (60-73)	68 (62-76)	0.541
BMI (kg/m^2^), median (IQR)	25 (23-29)	23 (22-26)	26 (23-29)	24 (22-27)	0.578
<20	9 (9)	0 (0)	8 (11)	1 (5)	0.628
20-<25	33 (32)	2 (50)	21 (28)	10 (45)	
25-<30	35 (34)	1 (25)	27 (36)	7 (32)	
30+	16 (16)	0 (0)	14 (18)	2 (9)	
Not recorded	9 (9)	1 (25)	6 (8)	2 (9)	
Liver disease					
HCV	40 (39)	1 (25)	34 (45)	5 (23)	0.125
HBV	30 (29)	1 (25)	20 (26)	9 (41)	0.409
Alcohol	28 (27)	0 (0)	22 (29)	6 (27)	0.449
NASH	21 (21)	1 (25)	16 (21)	4 (18)	0.934
Cryptogenic	3 (3)	0 (0)	1 (1)	2 (9)	0.154
Multiple	24 (24)	0 (0)	20 (26)	4 (18)	0.385
Systemic therapy					0.441
Sorafenib	15 (15)	0 (0)	10 (13)	5 (23)	
Lenvatinib	40 (39)	2 (50)	29 (38)	9 (41)	
Atezolizumab–bevacizumab	40 (39)	2 (50)	33 (43)	5 (23)	
Other immunotherapy	7 (7)	0 (0)	4 (5)	3 (14)	
Nivolumab	5 (5)				
MiNA trial	1 (1)				
Pembrolizumab	1 (1)				
ECOG					0.002
0	44 (43)	2 (50)	37 (49)	5 (23)	
1	47 (46)	2 (50)	36 (47)	9 (41)	
2	10 (10)	0 (0)	3 (4)	7 (32)	
3	1 (1)	0 (0)	0 (0)	1 (5)	
CPS, median (IQR)	6 (5-7)	5.5 (5-6.5)	6 (5-6)	6 (5-7)	0.182
A	76 (75)	3 (75)	60 (79)	13 (59)	0.262
B	24 (24)	1 (25)	14 (18)	9 (41)	
C	2 (2)	0 (0)	2 (3)	0 (0)	
MELD	8 (7-11)	6.5 (6-8)	8 (7-10)	9.5 (7-11)	0.507
Thrombocytopenia	51 (50)	0 (0)	39 (51)	12 (55)	0.120
AFP (μg/l), median (IQR)	35 (6-557)	308 (3-696)	31 (8-248)	91 (5-2300)	0.626
BCLC stage					0.944
B	39 (38)	1 (25)	30 (39)	8 (36)	
C	60 (59)	3 (75)	44 (58)	13 (59)	
D	3 (3)	0 (0)	2 (3)	1 (5)	
ALBI grade					0.314
1	22 (22)	1 (25)	19 (25)	2 (9)	
2	69 (68)	2 (50)	51 (67)	16 (73)	
3	11 (11)	1 (25)	6 (8)	4 (18)	

AFP, α-fetoprotein; ALBI, albumin–bilirubin; BCLC, Barcelona Clinic Liver Cancer; BMI, body mass index; CPS, Child–Pugh score; ECOG, Eastern Cooperative Oncology Group; HBV, hepatitis B virus; HCV, hepatitis C virus; IQR, interquartile range; MELD, Model for End-stage Liver Disease; NASH, non-alcoholic steatohepatitis.

### Frailty

The median LFI was 4.13 (IQR 3.81-4.43, Supplementary Figure S1, available at https://doi.org/10.1016/j.esmogo.2024.100043). Most patients were pre-frail (75%), but more were frail (22%) than robust (4%). Median LFI among frail patients was 4.81 (IQR 4.64-5.12), pre-frail patients 4.06 (IQR 3.69-4.24), and robust patients 3.03 (IQR 2.99-3.12). Frail patients were similar to other patients in terms of age, sex, BCLC stage, Child–Pugh score, aetiology of liver disease, and type of systemic therapy (Table 1). There were no differences in specific biochemical parameters (albumin, bilirubin, international normalised ratio, platelets, creatinine) between frail and non-frail patients. However, frail patients were more likely to be ECOG 2 or 3 than pre-frail or robust patients (37% frail, 4% pre-frail, 0% robust, *P* = 0.002).

### Outcomes

#### Overall survival

There were 69 deaths by the end of follow-up, with a median survival of 11.8 months [95% confidence interval (CI) 9.1-13.7 months]. The LFI as a continuous variable was associated with overall survival on univariable analysis [hazard ratio (HR) for death 1.62, 95% CI 1.13-2.51, *P* = 0.019, Table 2]. Survival was also predicted on univariable analysis by Child–Pugh score, BCLC stage, AFP, and ALBI grade. The LFI remained a significant predictor of death on multivariable analysis [adjusted HR (aHR) 1.74, 95% CI 1.17-2.59, *P* = 0.006], with other significant variables Child–Pugh score and ALBI grade. There was no significant collinearity in the survival model.

**Table 2:** Univariable and multivariable predictors of overall survival

	Univariable analysis HR (95% CI)	*P*	Multivariable analysis aHR (95% CI)	*P*
Frailty: categorical		0.216		
Frail	1.55 (0.91-2.65)			
Pre-frail	1			
Robust	0.71 (0.22-2.30)			
**LFI**	**1.68(1.13-2.51)**	**0.011**	**1.74 (1.17-2.59)**	**0.006**
Sex (female)	1.11 (0.60-2.08)	0.744		
Age	1.00 (0.98-1.03)	0.987		
BMI	0.99 (0.94-1.05)	0.708		
ECOG		0.127		
0	1			
1	1.17 (0.69-1.97)			
2/3	2.23 (1.02-4.85)			
Liver disease		0.439		
HCV	1			
Alcohol	1.59 (0.61-4.09)			
NASH	1.19 (0.54-2.69)			
HBV	0.99 (0.47-2.10)			
Other/multiple	1.70 (0.88-3.29)			
**CPS**	**1.87 (1.56-2.24)**	**<0.001**	**1.48 (1.15-1.90)**	**0.002**
**ALBI grade**		**<0.001**		**0.015**
**1**	**1**		**1**	
**2**	**3.57 (1.60-7.92)**		**2.42 (1.06-5.56)**	
**3**	**17.75 (6.66-47.31)**		**6.84 (1.86-25.24)**	
Thrombocytopenia	1.00 (1.00-1.00)	0.214		
**BCLC stage**		**0.011**		
**BCLC B**	**1**			
**BCLC C**	**1.15 (0.70-1.91)**			
**BCLC D**	**12.62 (3.49-45.65)**			
**AFP**	**1.00 (1.00-1.00)**	**0.022**		
Systemic therapy		0.633		
Sorafenib	1.24 (0.65-2.36)			
Lenvatinib	1			
Atezolizumab–bevacizumab	0.80 (0.44-1.42)			
Other	0.78 (0.30-2.00)			

Bolded entries indicate statistically significant results. AFP, α-fetoprotein; ALBI, albumin–bilirubin; BCLC, Barcelona Clinic Liver Cancer; BMI, body mass index; CPS, Child–Pugh score; ECOG, Eastern Cooperative Oncology Group; HBV, hepatitis B virus; HCV, hepatitis C virus; LFI, Liver Frailty Index; NASH, non-alcoholic steatohepatitis.

Frailty as a categorical variable was not predictive of survival using the pre-defined categories of frail, pre-frail, and robust^8^ (Supplementary Figure S2, available at https://doi.org/10.1016/j.esmogo.2024.100043), or dichotomous combinations of these categories (i.e. frail versus non-frail, robust versus non-robust). Survival was no different between patients receiving immunotherapy versus TKI, including on restriction of analysis to frail and pre-frail patients (HR 0.68, 95% CI 0.41-1.16, *P* = 0.152, Supplementary Figure S3, available at https://doi.org/10.1016/j.esmogo.2024.100043).

**Figure 1:**
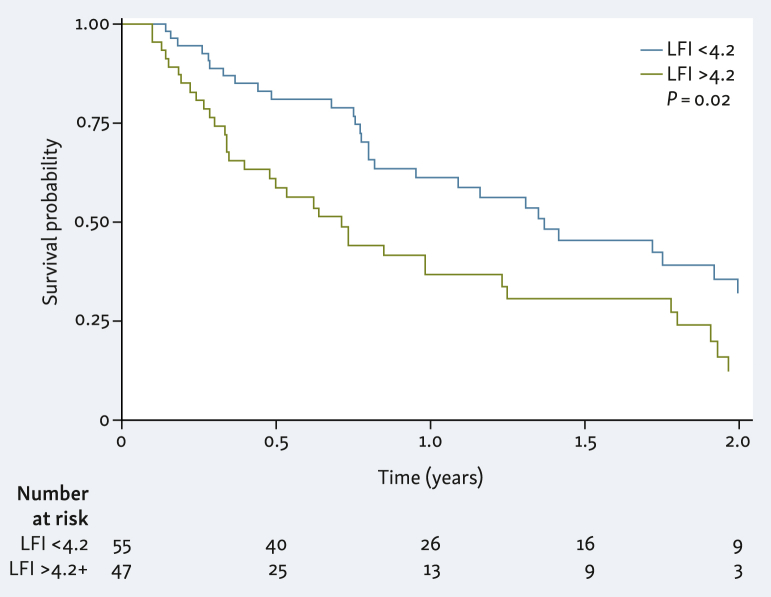
**Kaplan–Meier curve for survival by most predictive cut-off for frailty at 1 year (LFI 4.2)**. LFI, Liver Frailty Index.

The optimal cut-off of LFI to predict overall survival at 1 year was identified as an LFI of 4.2 (area under the curve 0.658, sensitivity 59%, and specificity 66%, Supplementary Figure S4, available at https://doi.org/10.1016/j.esmogo.2024.100043). This cut-off was significant at predicting death on both univariable and multivariable analyses (Figure 1, Supplementary Table S1, available at https://doi.org/10.1016/j.esmogo.2024.100043). Median survival was 8.5 months for patients more frail than the cut-off (95% CI 4.08-14.76 months) compared to 16.4 months for less frail patients (95% CI 9.8-23.9 months). The performance of other frailty cut-offs at discriminating survival is shown in Table 3.

**Table 3:** Median survival (months) for patients more frail and less frail than specific frailty cut-offs

LFI cut-off	LFI	*n* of more frail, less frail than cut-off	Median survival for those more frail than cut-off (95% CI)	Median survival for those less frail than cut-off (95% CI)	Log-rank *P*
90th percentile	4.84	11, 91	7.4 (2.2-21.6)	13.1 (9.2-17.0)	0.126
Frail according to original Lai et al*.* study^8^	4.5	22, 80	8.5 (3.2-21.4)	15.0 (9.6-21.0)	0.084
75th percentile	4.43	25, 77	8.8 (4.8-14.8)	15.0 (9.6-21.0)	0.079
**Optimal cut-off determined by ROC**	**4.2**	47, 55	8.5 (4.1-14.8)	16.4 (9.8-23.9)	**0.015**
**50th percentile**	**4.13**	52, 50	8.8 (5.8-11.8)	17.0 (11.4-23.9)	**0.025**
**25th percentile**	**3.81**	76, 26	9.2 (7.4-13.1)	21.0 (15.7-25.4)	**0.027**
10th percentile	3.34	90, 12	10.2 (8.5-15.7)	21.0 (9.8-25.4)	0.194
Robust according to original Lai et al. study^8^	3.2	98, 4	11.4 (9.0-16.2)	21.0 (17.0-25.4)	0.446

Bolded entries indicate statistically significant results. CI, confidence interval; LFI, Liver Frailty Index; ROC, receiver operator curve.

#### Secondary outcomes

Time to cessation of systemic therapy was correlated with frailty on univariable analysis as both a continuous (HR 1.73, 95% CI 1.15-2.62, *P* = 0.010) and a categorical variable (HR 2.35, 95% CI 1.36-4.07, *P* = 0.016, Table 4). Median time on systemic therapy was 2.4 months for frail patients (95% CI 1.0-3.1 months), 5.5 months for pre-frail (95% CI 3.4-8.2 months), and 5.4 months for robust patients (95% CI 2.6 months-not reached). Other significant factors on univariable analysis were Child–Pugh score, BCLC stage, thrombocytopenia, and ALBI grade. On multivariable analysis, frailty remained significant as a categorical variable (aHR 2.08, 95% CI 1.20-3.60, *P* = 0.030), but lost significance as a continuous variable (aHR 1.47, 95% CI 0.99-2.19, *P* = 0.055).

**Table 4:** Univariable and multivariable predictors of time to cessation of systemic therapy

	Univariable analysis HR (95% CI)	*P*	Multivariable analysis 1 aHR (95% CI)	*P*	Multivariable analysis 2 aHR (95% CI)	*P*
**Frailty**		**0.016**		**0.030**		
**Frail**	**2.35 (1.36-4.07)**		**2.08 (1.20-3.60)**			
**Pre-frail**	**1**		**1**			
**Robust**	**1.24 (0.45-3.45)**		**1.47 (0.53-4.10)**			
**Frailty score**	**1.73 (1.15-2.62)**	**0.010**			1.47 (0.99-2.19)	0.055
Sex (female)	1.36 (0.76-2.44)	0.327				
Age	0.99 (0.97-1.01)	0.418				
BMI	0.98 (0.93-1.03)	0.347				
ECOG		0.100				
0	1					
1	1.40 (0.87-2.24)					
2/3	2.24 (1.05-4.76)					
Liver disease		0.520				
HCV	1					
Alcohol	1.40 (0.59-3.32)					
NASH	0.94 (0.48-1.87)					
HBV	1.12 (0.57-2.19)					
Other/multiple	1.57 (0.87-2.85)					
**CPS**	**1.53 (1.26-1.85)**	**<0.001**	**1.50 (1.24-1.83)**	**<0.001**		
**ALBI grade**						**<0.001**
**1**	**1**	**<0.001**			**1**	
**2**	**1.95 (1.06-3.57)**				**1.83 (0.99-3.37)**	
**3**	**8.21 (3.34-20.17)**				**7.12 (2.85-17.86)**	
**Thrombocytopenia**	**1.00 (0.99-1.00)**	**0.030**				
**BCLC stage**						
**BCLC B**	**1**	**0.029**				
**BCLC C**	**1.16 (0.73-1.85)**					
**BCLC D**	**8.51 (2.38-30.43)**					
AFP	1.00 (1.00-1.00)	0.324				
Systemic therapy		0.085				
Sorafenib	1.35 (0.72-2.51)					
Lenvatinib	1					
Atezolizumab–bevacizumab	0.60 (0.36-1.01)					
Other	0.82 (0.35-1.96)					

Bolded entries indicate statistically significant results. AFP, α-fetoprotein; ALBI, albumin–bilirubin; BCLC, Barcelona Clinic Liver Cancer; BMI, body mass index; CPS, Child–Pugh score; ECOG, Eastern Cooperative Oncology Group; HBV, hepatitis B virus; HCV, hepatitis C virus; LFI, Liver Frailty Index; NASH, non-alcoholic steatohepatitis.

Reasons for cessation of systemic therapy were similar among groups by frailty categories (Supplementary Table S2, available at https://doi.org/10.1016/j.esmogo.2024.100043), including cessation due to disease progression or side-effects, although cessation due to functional decline was more common among frail (32%) than pre-frail (9%) or robust (0%) patients (*P* = 0.031).

Among the 80 patients who stopped the initial systemic therapy, over half of them (44, 55%) had further treatment, including second-line TKIs (30%), immunotherapy (11%), and stereotactic body radiotherapy (10%, Supplementary Table S3, available at https://doi.org/10.1016/j.esmogo.2024.100043). No frail patients were commenced on second-line immunotherapy (compared to 12% pre-frail and 50% robust patients, *P* = 0.014). Otherwise, the rates and type of further treatment did not differ between frail, pre-frail, and robust patients.

Adverse effects from systemic therapy were common among all patients (78% any adverse effect, 19% grade 3-4 adverse effects, Supplementary Table S4, available at https://doi.org/10.1016/j.esmogo.2024.100043). However, frailty categories were not a significant predictor of the frequency, severity, or type of adverse effects, including further dichotomous groupings (frail versus pre-frail and robust; frail and pre-frail versus robust).

LFI was not a significant predictor of disease progression on univariable or multivariable analysis (Supplementary Table S5, available at https://doi.org/10.1016/j.esmogo.2024.100043). Type of systemic therapy was the only significant predictor of disease progression, particularly sorafenib compared to lenvatinib (aHR 2.61, 95% CI 1.28-5.31, *P* = 0.015).

#### Sensitivity analysis

Sensitivity analysis excluding patients with Child–Pugh B9 or higher, or ECOG 3, excluded seven patients, but LFI (as a continuous variable) remained significantly associated with death and systemic therapy cessation.

#### Sarcopenia

Baseline sarcopenia measurements were available with contemporaneous cross-sectional imaging for 65 patients (64%). Of these, most (40/65, 62%) were sarcopenic. Sarcopenic patients had lower median BMI than non-sarcopenic patients [24 kg/m^2^ (21-26 kg/m^2^) versus 26 kg/m^2^ (26-32 kg/m^2^), *P* = 0.001], but other characteristics were similar (Supplementary Table S6, available at https://doi.org/10.1016/j.esmogo.2024.100043). Similar proportions of sarcopenic patients were robust, pre-frail, or frail (*P* = 0.372), but the median frailty score was slightly higher [4.2 (3.8-4.5) versus 4.0 (3.4-4.3), *P* = 0.045] in sarcopenic compared to non-sarcopenic patients. Spearman’s rank correlation found frailty score and sarcopenia to be independent (*P* = 0.070).

Sarcopenia as a categorical variable was not associated with survival (HR 1.08, 95% CI 0.58-2.00, *P* = 0.811), disease progression (HR 1.23, 95% CI 0.64-2.32, *P* = 0.525), or cessation of systemic therapy (HR 1.23, 95% CI 0.65-2.32, *P* = 0.525). There were no differences in the occurrence of any adverse events, severity of adverse events, or specific adverse events by sarcopenia. Analysis of sarcopenia as a continuous variable also showed no significant findings.

## Discussion

This prospective study is the first to investigate the relationship of frailty to outcomes in patients with HCC undergoing systemic therapy. We found that frailty (as determined by LFI) was common and an independent predictor of overall survival for patients receiving systemic therapy for HCC. In particular, this association was independent of ECOG, the traditional measure of performance status in HCC. Frailty was also associated with early discontinuation of therapy, but this was not secondary to disease progression or greater adverse events. Few patients in this cohort were robust (4%), and the median frailty (4.1) was higher than in cohorts of patients with cirrhosis awaiting LT (3.6, 3.8, 4.0).^8^^,^^10^^,^^26^ The LFI was also higher than reported among HCC patients awaiting LT (median LFI 3.2).^18^ Meanwhile, sarcopenia, although prevalent, was not similarly predictive of any outcome in this study.

Despite the significance of the LFI as a continuous variable, existing categories for frailty were not helpful in this cohort, as they were not predictive of survival on univariable or multivariable analysis. This is perhaps explained by the fact that the cut-offs were established among end-stage liver disease patients without HCC awaiting LT. Interestingly, these existing categories were independently predictive of time to cessation of systemic therapy suggesting that frailty may impact various HCC events differently and the need for different cut-offs. Within the limits of our relatively small study, we found a significantly shorter median overall survival [8.5 months (95% CI 4.1-14.8 months) versus 16.4 months (95% CI 9.8-23.9 months)] in those who were ‘more frail’ versus ‘less frail’ using our optimal LFI cut-off of 4.2. This raises the question of whether a futility cut-off to identify patients ‘too frail’ to benefit from systemic therapy could be demonstrated in studies with greater power. Frailty could be a promising factor used in patient selection in the future, perhaps adding to the discriminatory power of cancer stage and liver function.

The earlier cessation of systemic therapy among frailer patients (those with higher LFI) requires further clarification. Whether the less time on systemic therapy leads to worse survival in frailer patients or earlier death (and hence clinical deterioration) of frailer patients leads to earlier systemic therapy cessation is unclear. We did not find an increase in adverse events or disease progression in frailer patients to explain therapy cessation. This perhaps gives clinicians more confidence in prescribing systemic therapy in frail patients. Although interpretation is limited by low numbers, a higher proportion of frail patients ceased therapy due to functional decline, which could reflect worsening liver function or other comorbidities in these patients.

Whether frailty is modifiable in advanced HCC is unclear. Frailty is considered modifiable among older adults through physical interventions,^27^^,^^28^ and a consensus statement on frailty in cirrhosis suggests that the physical components of frailty should be considered modifiable, but evidence is limited.^29^ One study has examined in-hospital exercise on inpatients with HCC and demonstrated a 20-40-min programme per day improved the LFI compared to patients on bedrest.^30^ Given the short survival of patients with HCC commencing systemic therapy, any frailty intervention may need to start at earlier stages of HCC.

The strengths of this study include the multicentre and prospective design. The diverse aetiologies of liver disease and systemic treatments add to the generalisability of the results. The findings are limited by relatively low numbers, which also limited the ability to carry out subgroup analyses. The heterogeneous cohort included a few patients who were BCLC D, although excluding these patients from analysis did not significantly alter results. Clinicians were not blinded to findings, which may have led to differences in treatment of frail patients. Although the LFI has been shown to be reproducible in cirrhosis cohorts,^26^ it is not validated in HCC, which was measured here at treatment commencement only. One advantage of the LFI is that it is an easy and cheap assessment to carry out compared to other laboratory or imaging biomarkers used to prognosticate HCC. This may be of particular value in resource-limited countries where HCC is prevalent.

Future studies should validate these results with larger cohorts and identify HCC-specific cut-offs for frailty. Studies could consider whether change in frailty over time is more prognostic. Interventional studies of targeted (p)rehabilitation to reverse frailty during systemic therapy would be of great interest.

## Conclusion

Frailty, measured by LFI, is common among patients undergoing systemic therapy for HCC, and is an independent predictor of survival. LFI also predicts cessation of systemic therapy, but not adverse events or disease progression. Existing LFI cut-offs derived from transplant waitlist patients with HCC were not predictive in this cohort. The potential to modify frailty status with nutrition and rehabilitation intervention is an exciting area of future study.

## Data Sharing

Data are not publicly available due to ethical constraints, but are available on reasonable request to corresponding author.
