# Supplementary material for: The Liver Frailty Index predicts survival in systemic therapy for hepatocellular carcinoma: a multicentre prospective cohort study
Source: ESMO Gastrointest Oncol. 2024 Feb 20;3:100043. doi: 10.1016/j.esmogo.2024.100043 (PMC12836669; doi:10.1016/j.esmogo.2024.100043)
Supplement: Supplementary Material [file mmc1.docx]

Supplementary Figure 1: Distribution of frailty scores for all patients commencing systemic therapy

Supplementary Figure 2: Kaplan Meier curve for survival by existing cutoffs for frailty

Supplementary Figure 3: Kaplan Meier curve for survival by treatment choice (tyrosine kinase inhibitor vs immunotherapy) among frail and pre-frail patients

TKI: Tyrosine kinase inhibitor

Supplementary Figure 4: Receiver operator curve for survival at 1 year

Supplementary Table 1: Summary of outcomes using Liver Frailty Index score cutoff of 4.2

| **Outcome** | **UV HR** | **p** | **MV aHR** | **p** | **Other significant factors on MV analysis (p value)** |
| --- | --- | --- | --- | --- | --- |
| Death | 1.15 (1.03-1.29) | 0.016 | 1.15 (1.02-1.31) | 0.023 | ECOG (0.019) CPS (<0.001), AFP 0.032 |
| Disease progression | 1.07 (0.95-1.21) | 0.248 | 1.05 (0.93-1.19) | 0.43 | Systemic therapy type (0.020) |
| Systemic therapy cessation | 1.12 (1.01-1.24) | 0.042 | 1.08 (0.97-1.21) | 0.152 | ALBI (0.001) |

Abbreviations: aHR: adjusted Hazard Ratio, AFP Alpha fetoprotein, ALBI: Albumin-bilirubin, CPS: Child Pugh score, ECOG: Eastern Cooperative Oncology Group, HR: Hazard ratio, MV: Multivariable, UV: Univariable

Supplementary Table 2: Reasons for cessation of systemic therapy based on frailty status

| **Reason for cessation** | Total | Robust | Pre-frail | Frail | P value |
| --- | --- | --- | --- | --- | --- |
| Any cessation | 80 (78) | 4 (100) | 57 (75) | 19 (86) | 0.294 |
| Patient preference | 5 (6) | 0 (0) | 5 (9) | 0 (0) | 0.341 |
| Disease progression | 35 (44) | 3 (75) | 26 (46) | 6 (32) | 0.245 |
| Adverse effects | 34 (43) | 1 (25) | 24 (42) | 9 (47) | 0.709 |
| Decompensation | 9 (11) | 0 (0) | 8 (14) | 1 (5) | 0.442 |
| **Functional decline** | **11 (14)** | **0 (0)** | **5 (9)** | **6 (32)** | **0.031** |

Supplementary Table 3: Subsequent treatment post cessation of systemic therapy based on frailty status

|  | **Total** | **Robust** | **Pre-frail** | **Frail** | **p value** |
| --- | --- | --- | --- | --- | --- |
| Stopped treatment | 80 | 4 | 57 | 19 |  |
| Commenced new therapy | 44 (55) | 4 (100) | 30 (53) | 10 (53) | 0.179 |
| Tyrosine kinase inhibitor | 24 (30) | 2 (50) | 16 (28) | 6 (32) | 0.642 |
| **Immunotherapy** | **9 (11)** | **2 (50)** | **7 (12)** | **0 (0)** | **0.014** |
| Stereotactic body radiotherapy | 1 (1) | 1 (5) | 0 (0) | 0 (0) | 0.197 |
| Locoregional therapy | 8 (10) | 0 (0) | 7 (12) | 1 (5) | 0.536 |
| Other | 3 (4) | 0 (0) | 3 (5) | 0 (0) | 0.533 |

Supplementary Table 4: Adverse effects from systemic therapy based on frailty status

|  | **Total** | **Robust** | **Pre-frail** | **Frail** | **p value** |
| --- | --- | --- | --- | --- | --- |
| Any adverse effect | 80 (78) | 3 (75) | 62 (82) | 16 (73) | 0.648 |
| None | 21 (21) | 1 (25) | 16 (21) | 4 (18) | 0.899 |
| Grade 1 | 23 (23) | 0 (0) | 18 (24) | 5 (23) |  |
| Grade 2 | 39 (38) | 3 (75) | 27 (36) | 9 (41) |  |
| Grade 3 | 15 (15) | 0 (0) | 12 (16) | 3 (14) |  |
| Grade 4 | 4 (4) | 0 (0) | 3 (4) | 1 (5) |  |
| **Diarrhoea** | **22 (22)** | **0 (0)** | **21 (28)** | **1 (5)** | **0.038** |
| Hand-foot syndrome | 11 (11) | 0 (0) | 9 (12) | 2 (9) | 0.727 |
| Rash | 10 (10) | 1 (25) | 8 (11) | 1 (5) | 0.411 |
| Hypertension | 17 (17) | 0 (0) | 13 (17) | 4 (18) | 0.655 |
| Pain | 11 (11) | 0 (0) | 9 (12) | 2 (9) | 0.727 |
| Nausea | 14 (14) | 0 (0) | 10 (13) | 4 (18) | 0.599 |
| Lethargy | 11 (11) | 0 (0) | 9 (12) | 2 (9) | 0.727 |
| Weight loss | 5 (5) | 1 (25) | 3 (4) | 1 (5) | 0.164 |
| Anorexia | 9 (9) | 0 (0) | 7 (9) | 2 (9) | 0.817 |
| Wound issues | 7 (7) | 0 (0) | 3 (4) | 4 (18) | 0.057 |
| Hypothyroidism | 4 (4) | 1 (25) | 3 (4) | 0 (0) | 0.06 |
| GI bleeding | 5 (5) | 0 (0) | 5 (7) | 0 (0) | 0.407 |
| Proteinuria | 1 (1) | 0 (0) | 1 (1) | 1 (5) | 0.604 |
| Other side effects | 9 (9) | 1 (25) | 4 (5) | 4 (18) | 0.087 |

Supplementary Table 5: Univariable and multivariable predictors of disease progression according to RECIST 1.1

|  | **Univariable analysis  (HR, 95% CI)** | **p** | **Multivariable analysis**  **(aHR, 95% CI)** | **p** |
| --- | --- | --- | --- | --- |
| Frailty |  | 0.859 |  |  |
| Frail | 1.17 (0.63-2.18) |  |  |  |
| Pre-frail | 1 |  |  |  |
| Robust | 1.27 (0.45-3.57) |  |  |  |
| LFI | 1.12 (0.72-1.76) | 0.613 | 1.13 (0.71-1.81) | 0.600 |
| Sex (female) | 1.25 (0.63-2.48) | 0.540 |  |  |
| Age | 1.00 (0.97-1.03) | 0.963 |  |  |
| BMI | 0.96 (0.90-1.01) | 0.13 |  |  |
| ECOG |  | 0.133 |  |  |
| ECOG 0 | 1 |  |  |  |
| ECOG 1 | 1.19 (0.70-2.03) |  |  |  |
| ECOG 2/3 | 2.62 (1.11-6.22) |  |  |  |
| Liver disease type |  | 0.488 |  |  |
| HCV | 1 |  |  |  |
| Alcohol | 0.81 (0.27-2.45) |  |  |  |
| NASH | 1.07 (0.50-2.29) |  |  |  |
| HBV | 1.26 (0.60-2.65) |  |  |  |
| Other / Multiple | 1.77 (0.89-3.50) |  |  |  |
| CPS | 1.20 (0.94-1.52) | 0.164 |  |  |
| ALBI grade |  | 0.288 |  |  |
| 1 | 1 |  |  |  |
| 2 | 1.26 (0.70-2.27) |  |  |  |
| 3 | 2.39 (0.78-7.35) |  |  |  |
| Thrombocytopenia | 1.00 (1.00-1.00) | 0.854 |  |  |
| BCLC stage |  | 0.368 |  |  |
| BCLC B | 1 |  |  |  |
| BCLC C | 1.36 (0.80-2.30) |  |  |  |
| BCLC D | 3.24 (0.42-24.77) |  |  |  |
| AFP | 1.00 (1.00-1.00) | 0.836 |  |  |
| **Systemic therapy** |  | **0.022** |  | **0.015** |
| **Sorafenib** | **2.61 (1.28-5.30)** |  | **2.61 (1.28-5.31)** |  |
| **Lenvatinib** | **1** |  | **1** |  |
| **Atezolizumab-bevacizumab** | **1.03 (0.57-1.84)** |  | **1.06 (0.58-1.92)** |  |
| **Other** | **0.45 (0.14-1.52)** |  | **0.45 (0.13-1.50)** |  |

Abbreviations: AFP: Alpha fetoprotein, ALBI: Albumin-bilirubin, BCLC: Barcelona Clinic Liver Cancer, BMI: Body mass index, CPS: Child Pugh score, ECOG: Eastern Cooperative Oncology Group, HBV: Hepatitis B, HCV: Hepatitis C, LFI: Liver frailty index, NASH: Non-alcoholic steatohepatitis

Supplementary Table 6: Characteristics by sarcopenia status

| **Characteristics  (n, column %)** | **Total** | **Non-sarcopenic** | **Sarcopenic** | **p** |
| --- | --- | --- | --- | --- |
| Total | 65 | 25 | 40 |  |
| Frailty category |  |  |  |  |
| Robust | 3 (5) | 2 (8) | 1 (3) | 0.372 |
| Pre-Frail | 50 (77) | 20 (80) | 30 (75) |  |
| Frail | 12 (18) | 3 (12) | 9 (23) |  |
| Frailty score | 4.1 (3.6-4.4) | 4.0 (3.4-4.3) | 4.2 (3.8-4.5) | 0.045 |
| Sex |  |  |  | 0.811 |
| Male | 51 (78) | 20 (80) | 31 (78) |  |
| Female | 14 (22) | 5 (20) | 9 (23) |  |
| Age (median, IQR) | 65 (59-72) | 62 (57-68) | 66 (60-75) | 0.431 |
| BMI (median, IQR) | 25 (22-28) | 26 (26-32) | 24 (21-26) | 0.001 |
| <20 | 8 (13) | 3 (12) | 5 (13) | 0.016 |
| 20-<25 | 20 (31) | 3 (12) | 17 (44) |  |
| 25-<30 | 25 (39) | 11 (44) | 14 (36) |  |
| 30+ | 11 (17) | 8 (32) | 3 (8) |  |
| Not recorded |  |  |  |  |
| Liver disease |  |  |  |  |
| HCV | 32 (49) | 13 (52) | 19 (48) | 0.724 |
| HBV | 16 (25) | 7 (28) | 9 (23) | 0.617 |
| Alcohol | 19 (29) | 8 (32) | 11 (28) | 0.698 |
| NASH | 9 (14) | 3 (12) | 6 (15) | 0.733 |
| Cryptogenic | 1 (2) | 0 (0) | 1 (3) | 0.426 |
| Multiple |  |  |  |  |
| Systemic therapy |  |  |  | 0.717 |
| Sorafenib | 11 (17) | 5 (20) | 6 (15) |  |
| Lenvatinib | 30 (46) | 10 (40) | 20 (50) |  |
| Atezolizumab-bevacizumab | 24 (37) | 10 (40) | 14 (35) |  |
| ECOG |  |  |  | 0.571 |
| 0 | 27 (42) | 9 (36) | 18 (45) |  |
| 1 | 29 (45) | 12 (48) | 17 (43) |  |
| 2 | 8 (12) | 3 (12) | 5 (13) |  |
| 3 | 1 (2) | 1 (4) | 0 (0) |  |
| CPS (median, IQR) | 6 (5-7) | 6 (5-7) | 6 (5-7) | 0.539 |
| A | 47 (72) | 17 (68) | 30 (75) | 0.816 |
| B | 16 (25) | 7 (28) | 9 (23) |  |
| C | 2 (3) | 1 (4) | 1 (3) |  |
| MELD | 8 (7-10) | 8 (7-12) | 8 (7-10) | 0.783 |
| Thrombocytopenia | 34 (52) | 15 (60) | 19 (48) | 0.326 |
| AFP (ug/L, median, IQR) | 41 (5-557) | 45 (10-194) | 38 (4-1212) | 0.724 |
| BCLC stage |  |  |  | 0.404 |
| B | 26 (40) | 8 (32) | 18 (45) |  |
| C | 36 955) | 15 (60) | 21 (53) |  |
| D | 3 (5) | 2 (8) | 1 (3) |  |
| ALBI grade |  |  |  | 0.439 |
| 1 | 11 (17) | 6 (24) | 5 (13) |  |
| 2 | 47 (72) | 16 (64) | 31 (78) |  |
| 3 | 7 (11) | 3 (12) | 4 (10) |  |

Abbreviations: AFP: Alpha fetoprotein, ALBI: Albumin-bilirubin, BCLC: Barcelona Clinic Liver Cancer, BMI: Body mass index, CPS: Child Pugh score, ECOG: Eastern Cooperative Oncology Group, HBV: Hepatitis B, HCV: Hepatitis C, IQR: Inter-quartile range, MELD: Model for End Stage Liver Disease, NASH: Non-alcoholic steatohepatitis
